# Design and synthesis of tag-free photoprobes for the identification of the molecular target for CCG-1423, a novel inhibitor of the Rho/MKL1/SRF signaling pathway

**DOI:** 10.3762/bjoc.9.111

**Published:** 2013-05-21

**Authors:** Jessica L Bell, Andrew J Haak, Susan M Wade, Yihan Sun, Richard R Neubig, Scott D Larsen

**Affiliations:** 1 Vahlteich Medicinal Chemistry Core, Department of Medicinal Chemistry, College of Pharmacy, University of Michigan, Ann Arbor, MI 48109, USA; 2 Department of Pharmacology, University of Michigan Medical School, University of Michigan, Ann Arbor, MI 48109, USA; 3 College of Pharmacy, University of Michigan, Ann Arbor, MI 48109, USA

**Keywords:** CCG-1423, click ligation, photoaffinity labeling, Rho pathway inhibitor, tag-free photoprobe, target identification

## Abstract

CCG-1423 and related analogues represent a new class of inhibitors of Rho/MKL1/SRF-mediated gene transcription, a pathway that has been implicated in both cancer and fibrosis. The molecular target for these compounds is unknown. To facilitate its identification, a series of tag-free photoaffinity probes was designed and synthesized, each one containing a photoactivatable group and an acetylenic end group for subsequent attachment to a fluorescent tag using click chemistry. All were confirmed to maintain biological activity in a cell-based assay for inhibition of SRE-Luc expression. The functional activity of the most potent probe **24** was further confirmed in an assay for PC-3 cell migration. Photolysis of **24** in intact PC-3 cells followed by cell lysis, click ligation of a fluorescent dye, and gel electrophoresis revealed specific labeling of a single 24 kDa band that could be blocked with an active competitor. Future work will focus on identifying the labeled protein(s).

## Findings

Serum-induced signaling through Rho leads to gene-transcriptional effects, which are mediated by serum response factor (SRF), a MADS box transcription factor that binds to the serum response element (SRE) in the promoters of various immediate-early and muscle-specific genes. These signals involve release from cytosolic actin of the transcriptional coactivator MKL1, first identified in megakaryocytic leukemia (also known as myocardin-related transcription factor MRTF-A). Upon activation, MKL1 translocates to the nucleus where it binds to SRF to collaborate in the activation of the transcription of a number of genes including c-fos and SRF itself [1–4]. This pathway has been implicated in cancer metastasis [5] and, more recently, in fibrosis [6–7], making it an intriguing drug target.

**Figure 1:**
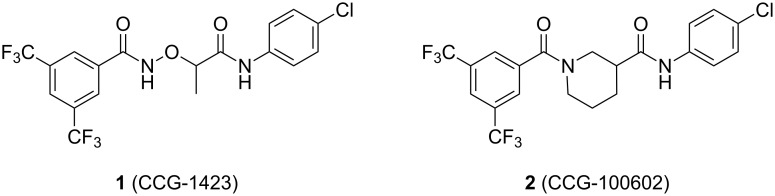
Structures of lead Rho/MKL1/SRF inhibitor **1** and conformationally restricted analogue **2**.

We recently reported a series of compounds that inhibit gene expression mediated by this Rho/MKL1/SRF signaling pathway [8]. The lead CCG-1423 (**1**, Figure 1) was identified in a cell-based high-throughput screen as an inhibitor of expression of a luciferase reporter gene driven by the serum response element promoter (SRE-Luc) [9]. Analysis of the mechanism of **1** showed that it acts at or downstream of MKL1 but upstream of SRF. Recent observations from our lab and others [10] have shown that **1** blocks MKL1 nuclear localization. The specific molecular target leading to inhibition of MKL1 function by **1**, however, is not known.

Identification of the macromolecular target(s) of **1** would allow us to employ rational and structure-based drug design to create more potent and selective therapeutics for the treatment of RhoA-related disorders. Early efforts at optimization in our laboratory led to nipecotic (bis)amide analogue **2** (CCG-100602, Figure 1), which was less potent (IC_50_ 9.8 µM versus 1.5 µM), but maintained similar maximal efficacy to that of **1** with significantly less cytotoxicity [8]. We therefore selected **2** as a template for the design of affinity probes.

The successful isolation/identification of molecular targets for low-affinity ligands and/or low-abundance targets requires photolabeling [11]. This permits extensive isolation and purification without premature dissociation from the target. Based on the structure–activity relationship (SAR) of **2**, we hypothesized that we could integrate photoactivatable functional groups without loss of activity. A variety of such groups that differ in their reactivities and chemoselectivities are available [12–13].

Photolabeling of cellular targets is typically done after cell lysis. However, if the molecular target for **2** is a multiprotein complex, membrane-bound, or integral to the nucleus, the process of cell lysis could compromise its integrity to such an extent that it would no longer retain its affinity for **2**. There are in fact reports of target ID studies that were only successful when performed using intact cells [14–16]. We considered this an ideal approach in light of our current hypothesis regarding the mechanism of action of **1** (redistribution of MKL primarily into the cytosol), which may require an intact actin cytoskeleton or nucleus. We thus elected to design photoaffinity probes that were tag-free, i.e., lacking either a biotin or fluorescent tag [17]. There are a number of distinct advantages to this approach: (1) the probes would retain low molecular weights and polar surface areas, and therefore good cell permeability, (2) minimal structural change would maximize the likelihood of maintaining affinity for the target; and (3) affinity for the target could be confirmed in cell-based phenotypic assays prior to any photolabeling studies*.* Tag-free photoprobes have also been reported to result in much less nonspecific binding relative to corresponding biotinylated photoprobes [18].

The design of our tag-free photoprobes followed the model pioneered by the Cravatt group in performing activity-based protein profiling (ABPP) [19–20]. This entails the incorporation of a ligand for recognition by the target, a reactive functionality for covalently bonding to the target, and either an azide or acetylene moiety as a latent linker for subsequent ligation of a tag for isolation and/or visualization (e.g., biotin or fluorophore) via click chemistry. This technology has been highly successful in profiling enzyme activity in living cells and even in whole organisms [21]. In ABPP, covalent linkage by the reactive functionality is usually dependent upon a particular enzymatic reaction, but photoactivatable groups (PGs) have also been used when this is not possible [22].

**Figure 2:**
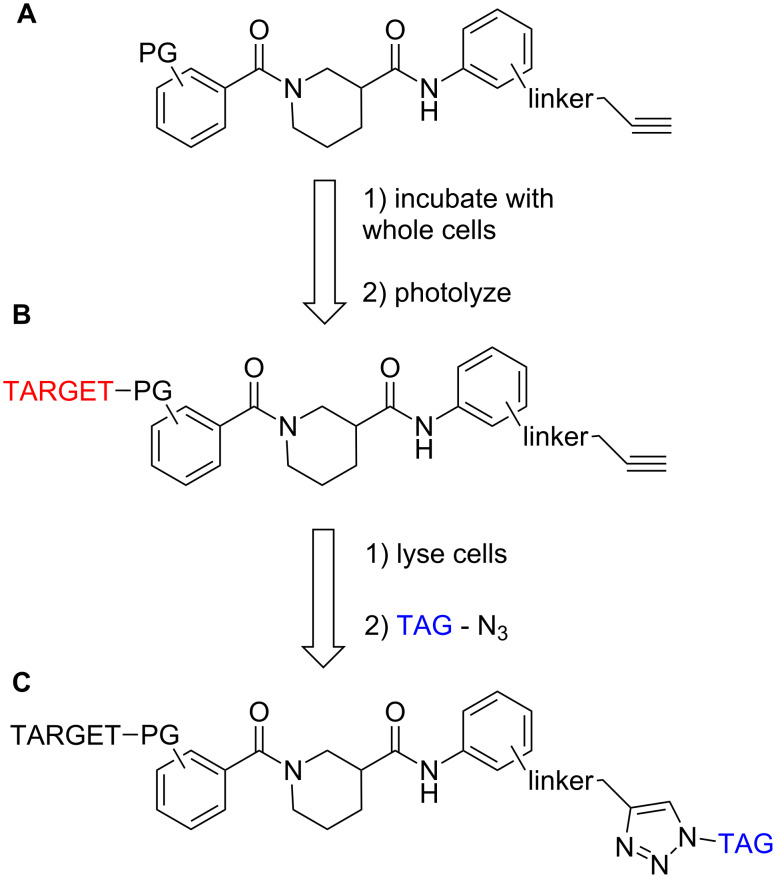
Strategy for tag-free photolabeling in whole cells (PG = photoactivatable group, TAG = fluorescent group or biotin).

We envisioned adapting this technology to intracellular target identification as depicted in Figure 2. Whole cells would be incubated with a photoprobe (A) after confirmation of its biological activity. Following exposure to UV light, the cells would be lysed, releasing the labeled proteins bound covalently to the probe (B). Click chemistry would then be applied to covalently attach a biotin or fluorescent tag for visualization and/or isolation (C). Any isolated proteins would be digested and identified by high-resolution mass-spectral analysis.

We selected benzophenone and azide as the PGs for incorporation into **2** based on synthetic ease and their complementary photoreactivities (benzophenone inserts into C–H bonds, azide into heteroatom–H bonds) [12]. Furthermore, we had previously established that benzophenone is tolerated on the benzamide ring with retention of the biological activity (**3**, Table 1) [23]. In addition to a PG, we needed a suitable linking functionality for attachment of a clickable acetylene group. Preliminary work (data not shown) established that an ether (versus amide) linker was superior with regard to maintenance of biological activity. A small set of model compounds was thus prepared to evaluate the impact of ether linker and PG placement on biological activity (see below in Table 1).

**Scheme 1:**
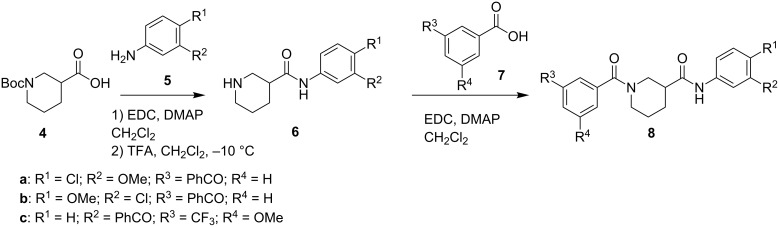
General synthesis of model benzophenone probes.

Scheme 1 depicts the general synthesis of model benzophenone-probes **8**. Boc-protected nipecotic acid (**4**) was reacted with substituted anilines **5** under standard EDC-mediated amidation conditions, followed by TFA-catalyzed deprotection to afford amides **6**. A subsequent second amidation with benzoic acids **7** afforded final bis(amide) analogues **8**.

**Scheme 2:**
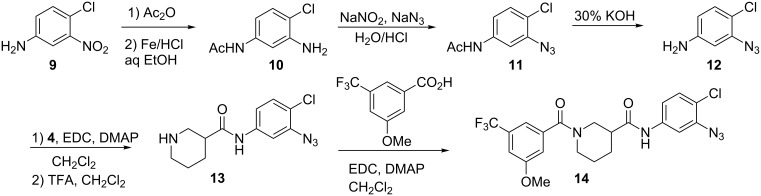
Synthesis of aryl azide model probe **14**.

We also synthesized a model azide (Scheme 2). Synthesis began with the acetylation of 4-chloro-3-nitroaniline (**9**) followed by reduction of the nitro group using iron and hydrochloric acid to generate aniline **10**. The azido group was introduced by diazotization/azidation to provide **11**. Deacetylation with potassium hydroxide revealed aniline **12**, which was then coupled with **4** and deprotected to give amine **13**. Final amidation with 3-methoxy-5-trifluoromethylbenzoic acid provided model probe **14**.

Table 1 presents biological activity data for all of the new model probes. These include: potency at inhibiting the Rho/MKL1/SRF-driven expression of a luciferase reporter gene (IC_50_ SRE.L) and a measure of maximal efficacy, as indicated by percent inhibition of SRE.L at the maximum dose tested (100 μM) [8–9]. We have noted in our previous work that both of these parameters are important for predicting activity in inhibiting cell migration [8]. As shown in Table 1, all of the model probes retained good activity relative to the lead compound **2** with little to no cytotoxicity. Based on these results we decided to install acetylenes in our final probes through ether linkages at the positions of the methoxy groups of the most active models **8a**, **8c** and **14**.

**Table 1:** Effects of model photoprobes on transcription and cytotoxicity in PC-3 cells^a^.

compound	structure	IC_50_ SRE.L (µM)^b^	% inhibition SRE.L (100 µM)^b^	% inhibition WST-1 (100 µM)^c^

**3**	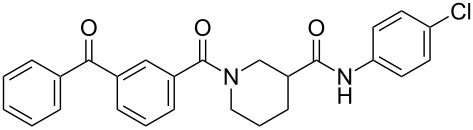	9.9	75	0
**8a**	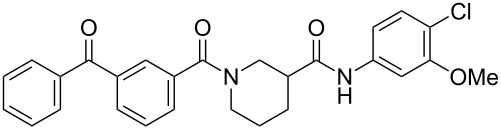	8.3	84	0
**8b**	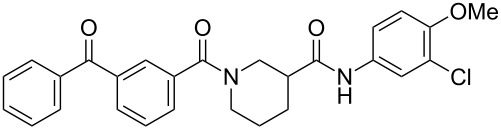	11	64	0
**8c**	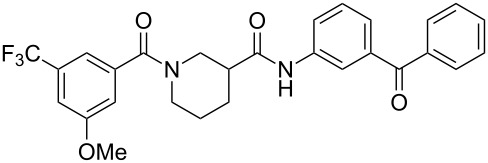	5.3	60	0
**14**	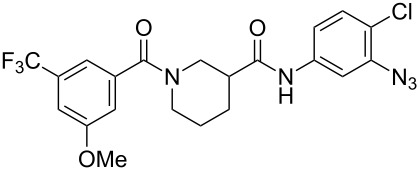	7.0	77	13

^a^For detailed assay descriptions, see Evelyn et al. [8–9]; ^b^Inhibition of Rho-pathway selective serum response element-luciferase reporter gene expression; ^c^Inhibition of mitochondrial metabolism of WST-1.

**Scheme 3:**
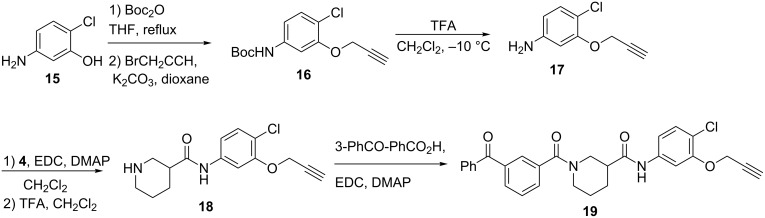
Synthesis of benzophenone photoaffinity probe **19**.

Preparation of the photoaffinity probe **19** is shown in Scheme 3. Aniline **15** was Boc-protected [24], and then alkylated using propargyl bromide to produce ether **16**. Following deprotection, aniline **17** was coupled with Boc-protected nipecotic acid (**4**), giving amide **18** after deprotection. Final coupling with 3-benzoylbenzoic acid afforded the final probe **19**.

**Scheme 4:**
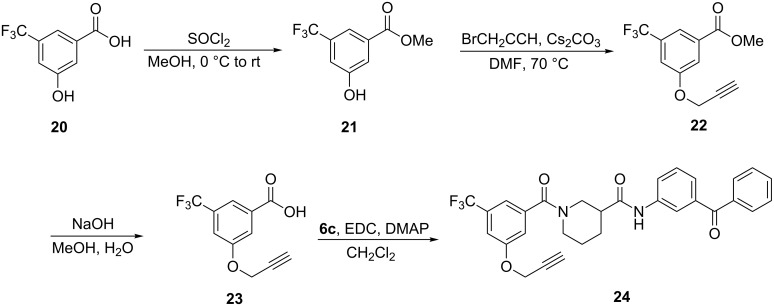
Synthesis of benzophenone photoaffinity probe **24**.

Synthesis of a photoprobe based on the most active model **8c** is shown in Scheme 4. Acid **20** was esterified prior to alkylation with propargyl bromide, affording ester **22**. Saponification, followed by amidation with the previously prepared piperidine **6c**, provided the final probe **24**.

**Scheme 5:**
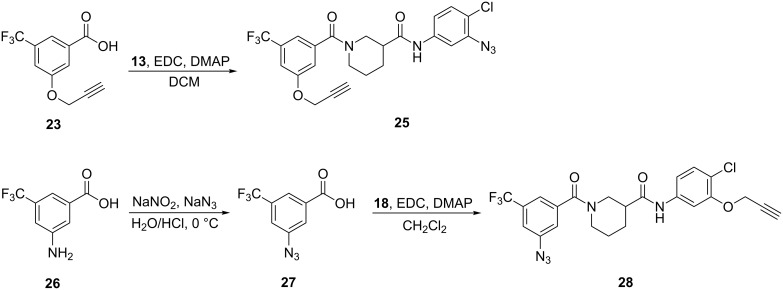
Synthesis of aryl azide photoaffinity probes.

Finally, two azide photoprobes were prepared. The first one, **25** (Scheme 5), based on model **14**, was synthesized in a straightforward manner by simply amidating acid **23** with previously prepared azide-containing piperdine **13** (Scheme 2). The second probe, **28**, reversing the placement of the azide and acetylene, was prepared from aniline **26**. Introduction of the azide was accomplished via diazotization/azidation, followed by standard amide coupling of **27** and **18** to afford final aryl azide photoprobe **28**.

Table 2 summaries the biological data for the four final candidate photoprobes. Fortuitously, all of the probes remained active in our assay, although the benzophenone analogues **19** and **24** were more potent than the azide probes **25** and **28**. Based on its superior potency, we selected benzophenone **24** (IC_50_ = 2.7 μM) for further studies.

**Table 2:** Effects of tag-free photoprobes on transcription and cytotoxicity in PC-3 cells^a^.

compound	structure	IC_50_ SRE.L (µM)	% inhibition SRE.L (100 µM)	% inhibition WST-1 (100 µM)

**19**	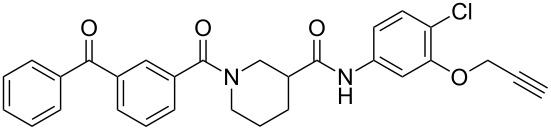	6.6	57	0
**24**	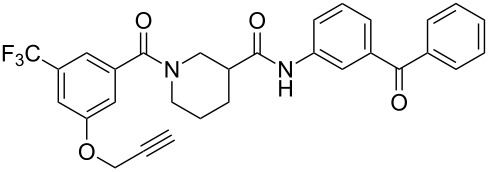	2.7	60	0
**25**	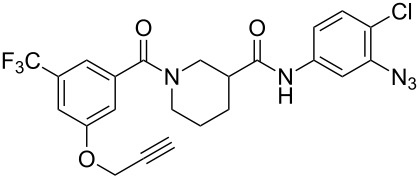	11	75	4
**28**	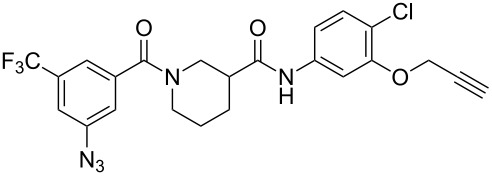	17	54	2

^a^See Table 1 for assay descriptions.

**Figure 3:**
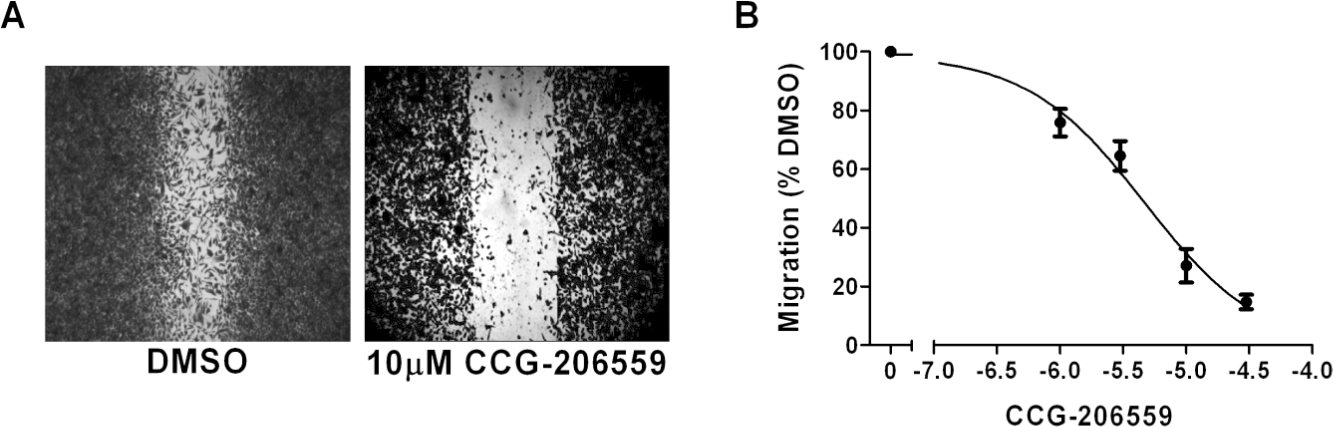
Photoprobe **24** (CCG-206559) retains biological activity to block prostate cancer migration. **A.** Cellular migration determined by wound assay. PC-3 prostate cancer cells were grown to confluence in 12-well plates and a scratch was made with a 200 µL pipette. Images were taken at the beginning of the experiment and after 24 h in DMEM containing 0.5% FBS and the indicated concentration of **24** or 0.1% DMSO. After 24 h the cells were stained with crystal violet to produce high-contrast images. Shown are examples of 10 μM **24** and DMSO after 24 h, from experiments with similar initial wound areas as determined by using image analysis software. **B.** Quantification of wound assay migration. The change in migration over 24 h was determined by the difference between the area of the wound after 24 h and the initial area of the wound. The percent inhibition was plotted by normalizing the compound-treated cells to the DMSO control. Results are expressed as the mean (±SEM) of triplicate experiments. A nonlinear least-squares regression curve was fit for CCG-206559 inhibition of migration, IC_50_ = 4.7 μM.

Prior to photolabeling, we wanted to confirm retention of functional activity in a PC-3 prostate cancer cell model of migration. Cells (5.0 × 10^5^) were plated in DMEM containing 10% FBS and grown to confluence in a 12-well plate. After 24 h, a scratch was made using a 200 µL pipette tip. Medium was replaced with DMEM containing 0.5% FBS and varying concentrations of **24** or 0.1% DMSO control. Images of the wound were taken at the beginning of the experiment by using a bright-field inverted microscope (Leica DM IRB) at 2.5× magnification. After 24 h the cells were fixed (10% formalin) and stained (0.5% crystal violet) to obtain high-contrast images. Area quantification of the wound was determined computationally using the ImageJ® software (NIH). The results are summarized in Figure 3. Probe **24** was clearly able to inhibit PC-3 cell migration into the scratch wound with an IC_50_ (4.7 μM) comparable to its potency at inhibiting SRE.L (2.7 μM).

**Figure 4:**
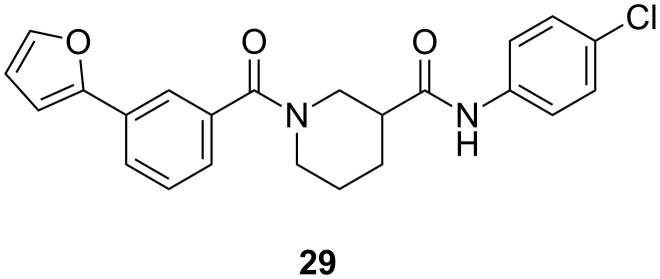
Structure of the competitor used in the photolabeling experiment.

Preliminary photolabeling studies with **24** were undertaken in PC-3 prostate cancer cells. Intact cells were treated with 0.3 µM **24** for 30 min. To facilitate the identification of specifically labeled proteins, a parallel competition experiment was also performed by treating cells with 0.3 µM **24** and a large excess (10 µM) of **29** (Figure 4), i.e., an analogue of **2** with slightly greater potency (IC_50_ = 6.4 µM) that was identified in later SAR studies [23]. UV irradiation was applied for 30 min at room temperature by using the long-wavelength setting of a hand-held illuminator (366 nm, UVL-56 lamp, UltraViolet Products, San Gabriel CA). Cells were then lysed and subsequently fluorescently tagged by using an Invitrogen^TM^ Click-iT^®^ Reaction Buffer Kit to attach azido-Cy5.5 dye for visualization.

**Figure 5:**
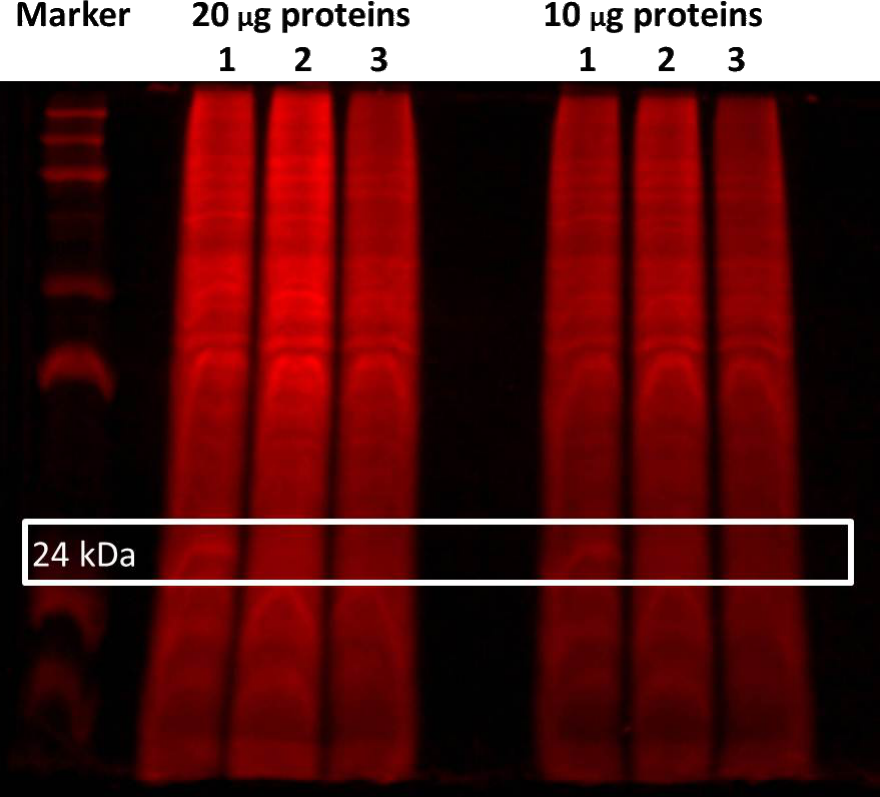
SDS-PAGE gel of photolabeling experiment in whole PC-3 cells. Lane 1 contains 0.3 µM **24** after 30 min UV treatment; lane 2 contains 0.3 µM **24** and 10 µM **29** after 30 min UV treatment; and lane 3 contains 0.3 µM **24** without UV treatment.

Electrophoresis of the tagged lysates was performed, using either 20 or 10 µg of protein, and is visualized in Figure 5. Lane 1 contains 0.3 µM of compound **24** alone, and lane 2 includes a much higher concentration of the competitor **29**. Lane 3 contains 0.3 µM of compound **24** alone without UV treatment. Although there is obviously a high degree of nonspecific binding under these conditions, a single band appears at approximately 24 kDa (as indicated by the white box) that was competed off by **29** and does not appear without UV treatment, suggesting that it is a specific binding protein that has been successfully photolabeled.

Although the level of fluorescent labeling in the non-UV-irradiated control lanes 3 is clearly diminished relative to lanes 1 and 2, it is nevertheless much higher than expected. Unfortunately, we do not yet have a satisfactory explanation for this. Future experiments will include using an even lower concentration of photoprobe (which is reported to minimize nonspecific binding [11]) and rigorous exclusion of light during processing of the cell lysates.

In conclusion, we have designed and synthesized tag-free benzophenone and aryl azide photoaffinity probes that retain the biological activity of the lead Rho/MKL1/SRF transcription inhibitor **2** in whole cells. Significantly, a preliminary photolabeling study with the most potent photoprobe **24** in whole prostate cancer cells was successful at detecting specific binding to one or more proteins at 24 kDa. Future work will focus on identifying the labeled protein(s). We first plan to repeat the photolabeling study with **24** and tag the labeled proteins by clicking with azido-biotin, thereby enabling isolations with streptavidin. Following purification, the 24 kDa region of the lanes (± competitor **29**) will be trypsin-digested and analyzed for differences by high resolution LC/MS/MS. If this is not successful, future work will entail using probe **24** and competitor **29** in SILAC experiments [25].

## Supporting Information

File 1 Full experimental synthetic procedures and spectral data are provided for compounds **4, 6a, 6b, 8a, 8b, 8c, 13, 14, 16–19, 21–25** and **28**.
