# Supplementary material for: Design and synthesis of tag-free photoprobes for the identification of the molecular target for CCG-1423, a novel inhibitor of the Rho/MKL1/SRF signaling pathway
Source: Beilstein J Org Chem. 2013 May 21;9:966–73. doi: 10.3762/bjoc.9.111 (PMC3678708; doi:10.3762/bjoc.9.111)
Supplement: File 1 — Full experimental synthetic procedures and spectral data are provided for compounds 4, 6a, 6b, 8a, 8b, 8c, 13, 14, 16–19, 21–25 and 28. [file Beilstein_J_Org_Chem-09-966-s001.pdf]

Supporting Information

for

Design and synthesis of tag-free photoprobes for the identification of the molecular target for CCG-1423, a novel inhibitor of the Rho/MKL1/SRF signaling pathway

Jessica L. Bell[1], Andrew J. Haak[2], Susan M. Wade[2], Yihan Sun[3], Richard R. Neubig[2] and Scott D. Larsen*[1]

Address: [1]Vahlteich Medicinal Chemistry Core, Department of Medicinal Chemistry, College of Pharmacy, University of Michigan, Ann Arbor, MI 48109, USA, [2]Department of Pharmacology, University of Michigan Medical School, University of Michigan, Ann Arbor, MI 48109, USA and [3]College of Pharmacy, University of Michigan, Ann Arbor, MI 48109, USA

Email: Scott D. Larsen* - sdlarsen@umich.edu

*Corresponding author

Full experimental synthetic procedures and spectral data are provided for compounds 4, 6a, 6b, 8a, 8b, 8c, 13, 14, 16–19, 21–25 and 28

Synthetic Procedures

All starting materials were obtained from commercial suppliers and were used without purification. All reactions involving air- or moisture-sensitive compounds were performed under an N_2 atmosphere. NMR spectra were recorded on a Bruker instrument at 500 MHz, a Bruker instrument at 300 MHz, or a Varian instrument at 400 MHz for ^1H. Chemical shift values are recorded in δ units (ppm). ESI mass spectra were recorded on a Waters Corporation LCT Time-of-Flight mass spectrometer or on an Agilent Technologies LC/MS system using a 1200 Series

LC and 6130 Quadrupole LC/MS (Agilent Technologies, Santa Clara, CA, USA) in positive mode. HPLC purities and retention times were recorded using an Agilent 1100 Series employing an Agilent ZORBAX Eclipse Plus C18 column (4.6 × 75 mm, 3.5 μm). Spectra were collected using Gradient A (10–90% acetonitrile/water over 13 min) or Gradient B (50–90% acetonitrile/water over 13 min) as indicated.

1-(*tert*-butoxycarbonyl)piperidine-3-carboxylic acid (4). Nipecotic acid (0.200 g, 1.549 mmol), and sodium hydroxide (1.703 mL, 1.703 mmol) were stirred in a solution of dioxane (3.40 mL) and water (1.70 mL). The solution was cooled to 0 °C and di-*tert*-butyl dicarbonate (0.395 mL, 1.703 mmol) was added. The reaction mixture was then warmed to room temperature and stirred overnight. The solution was concentrated in vacuo to approximately 2 mL and was cooled in an ice bath, covered with ca. 3 mL of ethyl acetate, and acidified with a dilute solution of $KHSO_4$ (10% solution) to pH ≈2. The aqueous phase was extracted with EtOAc (4 × 15 mL). The organics were combined, washed with brine (20 mL), dried ($MgSO_4$), filtered, and concentrated to afford 0.349 g (1.52 mmol, 96% yield) of the title compound as a white solid. No further purification was performed. ^1H NMR (500 MHz, DMSO-d_6) δ 12.38 (s, 1H), 4.03-3.76 (m, 1H), 3.75-3.60 (bs, 1H), 2.87-2.78 (m, 1H), 2.35-2.25 (m, 1H), 1.95-1.85 (m, 1H), 1.66-1.57 (m, 1H), 1.56-1.46 (m, 1), 1.42-1.28 (m, 11H).

General procedure for amide coupling (procedure A). Anhydrous dichloromethane (DCM) (1.0 mL) was added to a flask containing aniline (0.10 mmol) and was treated with EDC (0.15 mmol) followed by DMAP (0.15 mmol), and corresponding benzoic acid (0.11 mmol). The solution was stirred overnight at room temperature and was then diluted with DCM (4 mL) and was washed with 1M HCl (2.5 mL), followed by saturated $NaHCO_3$ (2.5 mL). The organics were dried ($MgSO_4$) and concentrated to afford a crude product, which was purified as needed by flash chromatography.

***tert*-butyl 3-((4-chloro-3-methoxyphenyl)carbamoyl)piperidine-1-carboxylate.** Prepared from **4** and 4-chloro-3-methoxyaniline according to procedure A. The crude material was purified using a CombiFlash system (35% EtOAc/Hex) to afford 0.139 g (0.377 mmol, 86% yield) of title compound as a clear oil. TLC R_f (50% EtOAc/Hex) 0.48. ^1H NMR (500 MHz, $CDCl_3$) δ 8.61

(bs, 1H), 7.62 (d, *J* = 2.4 Hz, 1H), 7.28-7.23 (m, 1H), 6.93 (s, 1H), 3.89 (s, 3H), 3.83-3.18 (m, 1H), 2.52 (s, 1H), 2.12 (s, 1H), 1.90 (s, 1H), 1.63 (s, 1H), 1.47 (s, 10H).

***N*-(4-chloro-3-methoxyphenyl)piperidine-3-carboxamide (6a).** *tert*-butyl 3-((4-chloro-3-methoxyphenyl)carbamoyl)piperidine-1-carboxylate (134 mg, 0.363 mmol) was dissolved in DCM (2.40 mL) and placed in an ice bath at approximately −5 °C. TFA (1.20 mL) was slowly added and the solution was stirred at −5 °C for 1 h. The solution was poured into 20 mL of cooled 2M NaOH. The mixture was extracted with DCM (3 × 20 mL), and the combined organic layers were dried ($MgSO_4$) and concentrated in vacuo to afford 0.073 g (0.27 mmol, 75% yield) of the title compound as a yellow solid. No further purification was performed. ^{1}H NMR (500 MHz, $CDCl_3$) δ 10.75 (s, 1H), 7.67 (d, *J* = 2.4 Hz, 1H), 7.28-7.23 (m, 1H), 6.94-6.82 (m, 1H), 3.92 (s, 3H), 3.36-3.22 (m, 1H), 3.13-3.05 (m, 1H), 2.95 (dd, *J* = 12.0, 3.2 Hz, 1H), 2.81-2.72 (m, 1H), 2.59-2.53 (m, 1H), 2.20-1.99 (m, 2H), 1.85-1.71 (m, 2H), 1.65-1.52 (m, 1H).

1-(3-benzoylbenzoyl)-*N*-(4-chloro-3-methoxyphenyl)piperidine-3-carboxamide (8a). Prepared from *N*-(4-chloro-3-methoxyphenyl)piperidine-3-carboxamide **6a** and 3-benzoylbenzoic acid according to procedure A, but using 1.1 equiv of EDC and DMAP. The crude material was triturated with DCM to afford 0.011 g (0.023 mmol, 17% yield) of the title compound as a white solid. ESI(+) *m*/*z*: $[M + H]^+$ 477.2; $[M + 23]^+$ 499.1. HPLC (gradient A): ret time = 7.36; purity: >95%. ^{1}H NMR (400 MHz, DMSO-d_6) 1.3:1.0 mixture of rotamers, δ 10.21 (rotamer 1, s), 10.01 (rotamer 2, s, 1H), 7.91-7.00 (m, 12H), 4.62-4.13 (m, 1H), 3.89-3.72 (m, 3H), 3.72-3.53 (m, 1H), 3.20-2.91 (m, 2H), 2.66-2.55 (m, 1H), 2.01 (s, 1H), 1.92-1.64 (m, 2H), 1.59-1.32 (m, 1H).

***tert*-butyl 3-((3-chloro-4-methoxyphenyl)carbamoyl)piperidine-1-carboxylate.** Prepared from **4** and 3-chloro-4-methoxyaniline according to Procedure A. The crude material was purified using a CombiFlash system (35% EtOAc/Hex) to afford 0.139 g (0.377 mmol, 86% yield) of the title compound as a clear oil. TLC R_f (50% EtOAc/Hex): 0.48. ^{1}H NMR (500 MHz, $CDCl_3$) δ 8.41 (s, 1H), 7.67 (s, 1H), 7.44 (dd, *J* = 8.8, 2.6 Hz, 1H), 6.85 (d, *J* = 8.8 Hz, 1H), 3.90-3.10 (m, 7H), 2.49 (s, 1H), 2.11 (s, 1H), 1.89 (s, 1H), 1.63 (s, 1H), 1.54-1.43 (m, 10H).

***N*-(3-chloro-4-methoxyphenyl)piperidine-3-carboxamide (6b).** *tert*-butyl 3-((3-chloro-4-methoxyphenyl)carbamoyl)piperidine-1-carboxylate (134 mg, 0.363 mmol) was dissolved in DCM (2.40 mL) and placed in an ice bath at approximately −5 °C. TFA (1.20 mL) was slowly added and the solution was stirred at −5 °C for 1 h. The solution was poured into 20 mL of cooled 2M NaOH. The mixture was extracted with DCM (3 × 20 mL), and the combined organic layers were dried ($MgSO_4$) and concentrated in vacuo to afford 0.063 g (0.234 mmol, 64% yield) of the title compound as a light brown solid. No further purification was performed. ^{1}H NMR (500 MHz, $CDCl_3$) δ 10.60 (s, 1H), 7.60 (d, *J* = 2.5 Hz, 1H), 7.50-7.48 (m, 1H), 6.87 (d, *J* = 8.8 Hz, 1H), 3.88 (s, 3H), 3.29-3.23 (m, 1H), 3.11-3.05 (m, 1H), 2.92 (dd, *J* = 11.9, 3.3 Hz, 1H), 2.73 (td, *J* = 10.9, 3.1 Hz, 1H), 2.53 (p, *J* = 3.6 Hz, 1H), 2.12-2.01 (m, 1H), 1.87 (s, 1H), 1.82-1.69 (m, 2H), 1.63-1.50 (m, 1H).

1-(3-benzoylbenzoyl)-*N*-(3-chloro-4-methoxyphenyl)piperidine-3-carboxamide (8b, 206118). Prepared from *N*-(3-chloro-4-methoxyphenyl)piperidine-3-carboxamide **6b** and 3-benzoylbenzoic acid according to procedure A, but using 1.1 equiv of EDC and DMAP. Crude material was purified using a CombiFlash system (65% EtOAc/Hex) to afford 0.040 g (0.084 mmol, 73% yield) of title compound as a yellow solid. ESI(+) *m*/*z*: $[M + H]^+$ 477.2; $[M + 23]^+$ 499.2. HPLC (gradient A): ret time = 7.17; purity: >95%. ^{1}H NMR (400 MHz, DMSO-d_6): 1.3:1.0 mixture of rotamers, δ 10.06 (rotamer 1, s), 9.87 (rotamer 2, s, 1H), 7.90-7.29 (m, 11H), 7.16-7.00 (m, 1H), 4.60-4.15 (m, 1H), 3.81 (s, 3H), 3.70-3.50 (m, 1H), 3.21-2.90 (m, 2H), 1.99 (bs, 1H), 1.90-1.65 (m, 2H), 1.58-1.30 (m, 1H).

***N*-(3-benzoylphenyl)-1-(3-methoxy-5-(trifluoromethyl)benzoyl)piperidine-3-carboxamide (8c, 206448).** Prepared as described above for **8a** and **8b** from **4** using 3-benzoylaniline and 3-methoxy-5-trifluoromethylbenzoic acid. Waters ESI(+) *m*/*z*: $[M + H]^+$ 511.3; $[M + Na]^+$ 533.3. HPLC (gradient A): ret time = 7.71 min; purity: >95%. ^{1}H NMR (400 MHz, DMSO-d_6) 1.3:1.0 mixture of rotamers, δ 10.31 (rotamer 1, s), 10.10 (rotamer 2, s, 1H), 8.10-7.18 (m, 12H), 4.58-4.10 (m, 1H), 3.93-3.75 (m, 3H), 3.63-3.40 (m, 1H), 3.17-2.93 (m, 2H), 2.70-2.55 (m, 1H), 2.08-1.97 (m, 1H), 1.90-1.65 (m, 2H), 1.57-1.35 (m, 1H).

***N*-(3-azido-4-chlorophenyl)piperidine-3-carboxamide (13).** 3-azido-4-chloroaniline **12** was prepared as described [1]. (0.075 g, 0.445 mmol) was dissolved in DCM (4.45 mL), and to the solution was added EDC (0.128 g, 0.667 mmol) followed by DMAP (0.082 g, 0.667 mmol) and **4** (0.112 g, 0.489 mmol) under stirring. The mixture was stirred at room temperature under nitrogen overnight. Additional DCM (5.55 mL) was added to the solution, and it was washed with saturated aqueous $NaHCO_3$ (10 mL), 1M HCl (10 mL), and then brine (10 mL). Organics were dried ($MgSO_4$) and concentrated to afford 0.149 g (0.392 mmol, 88% yield) of the title compound as a fluffy yellow solid. No further purification was performed**.** The crude amide was dissolved in dichloromethane (2.60 mL) and cooled to −5 °C. TFA (1.30 mL) was added slowly to the cooled solution. The solution was stirred at −5 °C for 1 h and was then poured into 10 mL of cooled 2M NaOH. The cooled basic solution was extracted with DCM (3 × 10 mL), and the organic layers were combined, dried ($MgSO_4$), and concentrated in vacuo to afford 0.094 g (0.336 mmol, 86% yield) of the title compound as an orange solid. No further purification was performed. ^{1}H NMR (400 MHz, DMSO-d_6) δ 10.21 (s, 1H), 7.87 (d, *J* = 1.4 Hz, 1H), 7.40 (d, *J* = 8.7 Hz, 1H), 7.33 (dd, *J* = 8.8, 2.0 Hz, 1H), 3.06-2.98 (m, 1H), 2.89-2.81 (m, 1H), 2.65-2.54 (m, 1H), 2.50-2.35 (m, 2H), 1.93-1.83 (m, 1H), 1.66-1.49 (m, 2H), 1.45-1.30 (m, 1H).

***N*-(3-azido-4-chlorophenyl)-1-(3-methoxy-5-(trifluoromethyl)benzoyl)piperidine-3-carboxamide (14).** Prepared from **13** and 3-methoxy-5-trifluoromethylbenzoic acid using procedure A. Agilent ESI(+) *m/z*: $[M + H]^+$ 482.0; $[M + Na]^+$ 504.0. HPLC (gradient A): ret time = 8.01 min; purity: >95%. ^{1}H NMR (400 MHz, DMSO-d_6) 1.3:1.0 mixture of rotamers, δ 10.35 (rotamer 1, s), 10.13 (rotamer 2, s, 1H), 8.00-7.66 (m, 1H), 7.50-7.12 (m, 5H), 4.58-4.00 (m, 1H), 3.90-3.77 (m, 3H), 3.63-3.69 (m, 1H), 3.09 (d, *J* = 37.6 Hz, 2H), 2.70-2.55 (m, 1H), 2.07-1.96 (m, 1H), 1.91-1.65 (m, 2H), 1.60-1.37 (m, 1H).

***tert*-butyl (4-chloro-3-(prop-2-yn-1-yloxy)phenyl)carbamate (16).** Aniline **15** was Boc-protected as described [2]. To a mixture of this compound (0.144 g, 0.591 mmol) and potassium carbonate (0.245 g, 1.773 mmol) in dioxane (1.45 mL) was added 3-bromoprop-1-yne (0.111 mL, 1.477 mmol). The reaction mixture was then heated to 80 °C for 48 h in a sealed tube. The reaction mixture was concentrated and was diluted with water (10 mL). The aqueous

layer was extracted with DCM (3 × 10 mL). The organics were washed with water (2 × 10 mL), dried ($MgSO_4$) and concentrated. Crude material was purified using a 12 g SiliaSep cartridge and was separated using a Biotage unit. Eight fractions were collected at 20 mL/min using a threshold of 20 mAU for collection. The gradient employed was 100% hexanes for 3 column volumes (CVs); ramping 0–20% over 15 CVs; and holding at 20% for 5 CVs. Fractions 4–8 (R_f: 0.35 in 20% ethyl acetate in hexanes) were combined and concentrated to yield 0.108 g of the title compound as a white solid. ^{1}H NMR (400 MHz, DMSO-d_6) δ 9.51 (s, 1H), 7.44 (d, J = 1.9 Hz, 1H), 7.29 (d, J = 8.7 Hz, 1H), 7.06 (dd, J = 8.7, 1.9 Hz, 1H), 4.81 (d, J = 2.3 Hz, 2H), 3.63 (t, J = 2.3 Hz, 1H), 1.47 (s, 9H).

4-Chloro-3-(prop-2-yn-1-yloxy)aniline (17). Compound **16** (0.105 g, 0.373 mmol) was dissolved in dichloromethane (2.50 mL) and cooled to −10 °C. TFA (1.25 mL) was added slowly to the cooled solution. The solution was stirred at −5 °C for 45 min and was then poured into 10 mL of cooled 2M NaOH. The cooled basic solution was extracted with dichloromethane (3 × 10 mL), and the combined organics were dried ($MgSO_4$) and concentrated to afford 0.064 g (0.352 mmol) of the title compound as a yellow solid. No further purification was performed. ^{1}H NMR (400 MHz, DMSO-d_6) δ 6.99 (d, J = 8.5 Hz, 1H), 6.38 (d, J = 2.1 Hz, 1H), 6.17 (dd, J = 8.5, 2.2 Hz, 1H), 5.29 (s, 2H), 4.75 (d, J = 2.0 Hz, 2H), 3.61 (s, 1H).

***N*-(4-chloro-3-(prop-2-yn-1-yloxy)phenyl)piperidine-3-carboxamide (18).**
Compound **17** (0.061 g, 0.336 mmol) was dissolved in dichloromethane (3.36 mL), and to the solution was added EDC (0.097 g, 0.504 mmol) followed by DMAP (0.062 g, 0.504 mmol) and **4** (0.085 g, 0.369 mmol) under stirring. The mixture was stirred at room temperature under nitrogen overnight. Additional dichloromethane (6.7 mL) was added to the solution, and it was washed with saturated aqueous $NaHCO_3$ (10 mL), 1M HCl (10 mL), and then brine (10 mL) and was dried ($MgSO_4$) and concentrated to afford 0.109 g (0.277 mmol, 83% yield) of the title compound as a white solid. No further purification was performed. The crude amide (0.107 g, 0.272 mmol) was dissolved in dichloromethane (1.82 mL) and cooled to −5 °C. TFA (0.910 mL) was added slowly to the cooled solution. The solution was stirred at −5 °C. After 1 h, the solution was poured into 5 mL of cooled 2M NaOH. The cooled basic solution was extracted with dichloromethane (3 × 5 mL), and the organic layers were combined, dried ($MgSO_4$), and

concentrated to afford 0.050 g (0.171 mmol, 63% yield) of the title compound as a white solid. No further purification was performed. ^{1}H NMR (400 MHz, DMSO-d_6) δ 10.11 (s, 1H), 7.65-7.57 (m, 1H), 7.33 (d, J = 8.6 Hz, 1H), 7.22 (dd, J = 8.6, 1.7 Hz, 1H), 4.83 (d, J = 1.8 Hz, 2H), 3.64 (s, 1H), 3.06-2.99 (m, 1H), 2.90-2.83 (m, 1H), 2.66-2.57 (m, 6H), 2.48-2.38 (m, 2H), 1.92-1.83 (m, 1H), 1.68-1.50 (m, 2H), 1.45-1.33 (m, 1H).

1-(3-benzoylbenzoyl)-*N*-(4-chloro-3-(prop-2-yn-1-yloxy)phenyl)piperidine-3-carboxamide (19). Prepared using **18** and 3-benzoylbenzoic acid according to procedure A. The crude material was purified using a Biotage FlashMaster Personal^{+} system (30–100% EtOAc/Hex) to afford 0.049 g (0.098 mmol, 58% yield) of the title compound as a white solid. TLC R_f (50% EtOAc/Hex): 0.18. Agilent ESI(+) *m*/*z*: [M + H]$^{+}$ 501.0; [M + Na]$^{+}$ 523.0. HPLC (gradient A): ret time = 7.40 min; purity: >95%. ^{1}H NMR (400 MHz, DMSO-d_6) 1.4:1.0 mixture of rotamers, δ 10.22 (rotamer 1, s), 10.02 (rotamer 2, s, 1H), 7.98-7.44 (m, 10H), 7.40-7.03 (m, 2H), 4.87-4.75 (m, 1H), 4.58-4.14 (m, 1H), 3.76-3.49 (m, 2H), 3.17-2.83 (m, 2H), 1.98 (s, 1H), 1.89-1.60 (m, 2H), 1.57-1.31 (m, 1H).

Methyl 3-hydroxy-5-(trifluoromethyl)benzoate (21). 3-hydroxy-5-(trifluoromethyl)benzoic acid **20** (0.220 g, 1.067 mmol) was dissolved in methanol (1.60 mL) and cooled to 0 °C. Thionyl chloride (0.117 mL, 1.601 mmol) was added slowly. Following addition, the bath was allowed to warm to room temperature. Stirring of the reaction continued at room temperature overnight. The reaction was concentrated in vacuo and was dissolved in ethyl acetate (5 mL). The organic layer was then washed with saturated $NaHCO_3$ (5 mL), followed by brine (5 mL). The organic layer was then dried ($MgSO_4$), filtered, and concentrated to afford 0.193 g (0.877 mmol, 82% yield) of the title compound as an off-white solid. No further purification was performed. ^{1}H NMR (400 MHz, DMSO-d_6) δ 10.61 (s, 1H), 7.61-7.54 (m, 2H), 7.28 (t, J = 2.0 Hz, 1H), 3.84 (s, 3H).

Methyl 3-(prop-2-yn-1-yloxy)-5-(trifluoromethyl)benzoate (22). To a mixture of ester **21** (0.181 g, 0.822 mmol) and cesium carbonate (0.268 g, 0.822 mmol) in DMF (2.05 mL) was added 3-bromoprop-1-yne (0.155 mL, 2.06 mmol). The reaction mixture was then heated to 70 °C in a sealed tube. After stirring overnight, the reaction mixture was concentrated and was diluted with water (10 mL). The aqueous layer was extracted with DCM (3 × 10 mL). The

combined organics were washed with water (10 mL), dried ($MgSO_4$), and concentrated in vacuo to afford 0.154 g (0.596 mmol, 73% yield) of the title compound as a yellow oil. No further purification was performed. ^{1}H NMR (400 MHz, DMSO-d_6) δ 7.85-7.76 (m, 2H), 7.63 (t, *J* = 2.0 Hz, 1H), 5.03 (d, *J* = 2.3 Hz, 2H), 3.90 (s, 3H), 3.67 (t, *J* = 2.3 Hz, 1H).

3-(prop-2-yn-1-yloxy)-5-(trifluoromethyl)benzoic acid (23). A solution of sodium hydroxide (0.061 g, 1.521 mmol), methanol (1.20 mL), and water (0.400 mL) was added to a vial containing **22** (0.151 g, 0.585 mmol). The reaction was stirred for 3 h and was then concentrated in vacuo. The residue was acidified using 2N HCl (5 mL). The aqueous layer was extracted with DCM (3 × 5 mL). The combined organics were dried ($MgSO_4$) and concentrated to afford 0.107 g (0.438 mmol, 75% yield) of the title compound as a white solid. No further purification was performed. ^{1}H NMR (400 MHz, DMSO-d_6) δ 13.59 (s, 1H), 7.83-7.75 (m, 2H), 7.59 (t, *J* = 2.0 Hz, 1H), 5.01 (d, *J* = 2.3 Hz, 3H), 3.66 (t, *J* = 2.3 Hz, 1H).

***N*-(3-benzoylphenyl)-1-(3-(prop-2-yn-1-yloxy)-5-(trifluoromethyl)benzoyl)piperidine-3-carboxamide (24).** Prepared by general procedure A from **23** and **6c**. Agilent ESI(+) *m*/*z*: $[M + H]^+$ 535.1; $[M + Na]^+$ 557.0. HPLC (gradient A): ret time = 7.74 min; purity = 95%. ^{1}H NMR (400 MHz, DMSO-d_6) 1.6:1.0 mixture of rotamers, δ 10.28 (rotamer 1, s), 10.06 (rotamer 2, s, 1H), 8.11-7.22 (m, 12H), 5.00-4.85 (m, 1H), 4.55-4.08 (m,1H), 3.71-3.34 (m, 2H), 3.17-2.88 (m, 2H), 2.62-2.51 (m, 1H), 2.02-1.97 (m, 1H), 1.87-1.60 (m, 2H), 1.43 (s, 1H).

***N*-(3-azido-4-chlorophenyl)-1-(3-(prop-2-yn-1-yloxy)-5-(trifluoromethyl)benzoyl)piperidine-3-carboxamide (25).** Prepared using **23** and **13** according to procedure A. The crude material was purified using a Biotage FlashMaster Personal$^+$ (40% EtOAc/Hex) to afford 0.055 g (0.109 mmol, 42% yield) of the title compound as a pale yellow solid HPLC (gradient A): ret time = 8.01 min; purity: >95%. ESI(+) *m*/*z*: $[M + Na]^+$ 527.9. ^{1}H NMR (400 MHz, DMSO-d_6) 1.5:1.0 mixture of rotamers, δ 10.36 (rotamer 1, s), 10.14 (rotamer 2, s, 1H), 7.90-7.72 (m, 1H), 7.51-7.12 (m, 5H), 5.06-4.88 (m, 2H), 4.63-4.06 (m, 1H), 3.73-3.35 (m, 2H), 3.20-2.92 (m, 2H), 2.65-2.52 (m, 1H), 2.09-1.99 (m, 1H), 1.90-1.64 (m, 2H), 1.47 (s, 1H).

1-(3-azido-5-(trifluoromethyl)benzoyl)-*N*-(4-chloro-3-(prop-2-yn-1-yloxy)phenyl)piperidine-3-carboxamide (28). Prepared from **18** and 3-azido-5-trifluoromethylbenzoic acid [3] using procedure A. Agilent ESI(+) *m*/*z*: $[M + H]^+$ 506.0; $[M+Na]^+$ 528.0. HPLC (gradient A): ret time = 7.75 min; purity: >90%. ^{1}H NMR (400 MHz, DMSO-d_6) 1.2:1.0 mixture of rotamers, δ 10.24 (rotamer 1, s), 10.02 (rotamer 2, s, 1H), 7.68-6.99 (m, 6H), 4.90-4.75 (m, 2H), 4.58-4.07 (m, 1H), 3.58-3.40 (m, 2H), 3.21-2.89 (m, 2H), 2.74-2.54 (m, 1H), 2.03 (s, 1H), 1.88-1.64 (m, 2H), 1.46 (bs, 1H).

References

1. Lamotte, H.; Degeilh, F.; Neau, P.; Ripoche, P.; Rousseau, B., *J. Labelled Compd. Radiopharm.* **1994,** *34*, 289–295.

2. Dhanak, D.; Knight, S. D. Sulfonamide derivative urotensin-II receptor antagonists, preparation, pharmaceutical compositions, and therapeutic use. WO2001045694A1, June 28, 2001.

3. Lang, H.-J.; Weichert, A.; Kleemann, H.-W.; Schwark, J.-R.; Scholz, W.; Albus,U. Preparation of substituted benzoylguanidiines as inhibitors of cellular Na+/H+ exchange. EP612723A1, Aug 31, 1994.
